# A Quantitative *Arabidopsis* IRE1a Ribonuclease-Dependent *in vitro* mRNA Cleavage Assay for Functional Studies of Substrate Splicing and Decay Activities

**DOI:** 10.3389/fpls.2021.707378

**Published:** 2021-07-20

**Authors:** Danish Diwan, Xiaoyu Liu, Caroline F. Andrews, Karolina M. Pajerowska-Mukhtar

**Affiliations:** Department of Biology, The University of Alabama at Birmingham, Birmingham, AL, United States

**Keywords:** *Arabidopsis thaliana*, *Nicotiana benthamiana*, unfolded protein response, IRE1, *bZIP60*, *in vitro* mRNA cleavage, ER stress, RNase-dead

## Abstract

The unfolded protein response (UPR) is an adaptive eukaryotic reaction that controls the protein folding capacities of the endoplasmic reticulum (ER). The most ancient and well-conserved component of the UPR is Inositol-Requiring Enzyme 1 (IRE1). Arabidopsis IRE1a (AtIRE1) is a transmembrane sensor of ER stress equipped with dual protein kinase and ribonuclease (RNase) activities, encoded by its C-terminal domain. In response to both physiological stresses and pathological perturbations, AtIRE1a directly cleaves *bZIP60* (*basic leucine zipper 60*) mRNA. Here, we developed a quantitative *in vitro* cleavage assay that combines recombinant AtIRE1a protein that is expressed in *Nicotiana benthamiana* and total RNA isolated from Arabidopsis leaves. Wild-type AtIRE1a as well as its variants containing point mutations in the kinase or RNase domains that modify its cleavage activity were employed to demonstrate their contributions to cleavage activity levels. We show that, when exposed to total RNA *in vitro*, the AtIRE1a protein cleaves *bZIP60* mRNA. Depletion of the bZIP60 transcript in the reaction mixture can be precisely quantified by a qRT-PCR-mediated assay. This method facilitates the functional studies of novel plant IRE1 variants by allowing to quickly and precisely assess the effects of protein mutations on the substrate mRNA cleavage activity before advancing to more laborious, stable transgenic approaches *in planta*. Moreover, this method is readily adaptable to other plant IRE1 paralogs and orthologs, and can also be employed to test additional novel mRNA substrates of plant IRE1, such as transcripts undergoing degradation through the process of regulated IRE1-dependent decay (RIDD). Finally, this method can also be modified and expanded to functional testing of IRE1 interactors and inhibitors, as well as for studies on the molecular evolution of IRE1 and its substrates, providing additional insights into the mechanistic underpinnings of IRE1-mediated ER stress homeostasis in plant tissues.

## Introduction

The endoplasmic reticulum (ER) is the largest cellular organelle that controls the fundamental part of the protein homeostasis network by folding nascent peptides, packaging them, and sending them to their target locations (Brandizzi, 2021; Howell, 2021; Verchot and Pajerowska-Mukhtar, 2021). ER also serves as a hub for cellular-adaptive responses to environmental challenges, including nutrient deficiency, oxidative or chemical stress, virus and bacterial infection, and heat. Various abiotic and biotic stresses cause numerous disruptions to protein folding in the ER, leading to the activation of unfolded protein response (UPR) (Afrin et al., 2020a; Pastor-Cantizano et al., 2020). In animals, plants, and yeast, the most conserved axis of UPR is Inositol-Requiring Enzyme 1 (IRE1), a hybrid kinase equipped with an N-terminal ER lumen sensor domain and a C-terminal endonuclease (RNase) domain (Chen and Brandizzi, 2013b; Ruberti and Brandizzi, 2014; Ruberti et al., 2015; Srivastava et al., 2018; Stefano and Brandizzi, 2018; Bao et al., 2019). The Arabidopsis genome encodes three isoforms of IRE1: AtIRE1a, AtIRE1b, and AtIRE1c (Koizumi et al., 2001; Mishiba et al., 2019; Pu et al., 2019). Although AtIRE1a and AtIRE1b have partly overlapping functions in UPR (Moreno et al., 2012; Chen and Brandizzi, 2013b; Afrin et al., 2020a; Verchot and Pajerowska-Mukhtar, 2021), the two isoforms show functional divergence and play distinct roles in response to various forms of stress. AtIRE1a is preferentially involved in pathogen-induced UPR whereas AtIRE1b shows higher activation when exposed to abiotic and chemical stress treatments, such as the *N*-glycosylation inhibitor tunicamycin (Tm) (Moreno et al., 2012). AtIRE1c lacks the N-terminal domain and was shown to be primarily involved in reproductive development (Mishiba et al., 2019; Pu et al., 2019).

The presumed mechanism of plant UPR activation involves IRE1 dimerization or oligomerization, *trans*-autophosphorylation of the cytoplasmic kinase domains, and activation of the C-terminal RNase domains (Ruberti et al., 2015; Mishiba et al., 2019; Li and Howell, 2021). The activated form of Arabidopsis IRE1 unconventionally splices mRNA for the *bZIP60un* (*basic leucine zipper 60*; unspliced) transcription factor (TF) in a process termed regulated IRE1-dependent splicing (RIDS). The spliced form of *bZIP60* (termed *bZIP60s*) gives rise to an active TF that translocates into the nucleus to drive the expression of ER stress-responsive genes (Deng et al., 2011; Moreno et al., 2012). Under acute or prolonged ER stress, eukaryotic IRE1 is also known to cleave and degrade specific mRNAs encoding proteins involved in the folding of nascent peptides and cell death through the regulated IRE1-dependent decay (RIDD) pathway. RIDD is activated to reduce the ER protein folding load or to initiate specific cytoprotective responses (Hollien and Weissman, 2006).

While RIDD has been documented to occur in plants and some of the Arabidopsis RIDD substrates have been identified, the precise mechanisms of plant RIDD and functions of the degraded mRNAs are not completely understood (Mishiba et al., 2013; Bao et al., 2018). In addition, global scale assays to discover potential IRE1 substrates, while undoubtedly useful, do not allow evaluating the direct engagement of IRE1 with the potential target mRNAs. Further, mutational studies of plant IRE1 homologs aiming to define the effects of individual subdomains and residues on the RNase activity levels are currently limited by the constraints related to the cost and time investment required to pursue stable transgenic approaches *in planta*. Working with non-model species further complicates such analyses due to technical limitations of transformation protocols (Hwang et al., 2017). Finally, the ER stress machinery represents a major intervention point for crop improvement because of the added value in protecting plants against heat and drought stress and supporting defenses against bacterial and fungal pathogens (Park and Park, 2019); however, limited efforts have been dedicated to date toward the development of methodologies focused on studies of plant ER stress responses (Chen and Brandizzi, 2013a; McCormack et al., 2015; Alcantara et al., 2020).

With these considerations in mind, we focused on the Arabidopsis IRE1a isoform and developed a quantitative *in vitro* mRNA cleavage assay. Our protocol employs *Nicotiana benthamiana*-expressed, recombinant AtIRE1a protein variants, and the well-established RIDS client *bZIP60un* mRNA. We included AtIRE1a variants containing point mutations in the kinase or RNase domains to experimentally test whether these protein variants exhibit diminished cleavage activity. Co-incubation of total RNA with AtIRE1a protein was followed by a qRT-PCR analysis to precisely quantify the depletion of *bZIP60un* mRNA that results from the AtIRE1a-mediated RIDS activity. While our method allowed us to characterize the cleavage efficacy of two novel AtIRE1a variants, this protocol can also be applied to the functional studies of other plant IRE1 paralogs, orthologs, and variants. Furthermore, it is readily adaptable to test additional novel mRNA substrates of plant IRE1, such as transcripts undergoing RIDD. Finally, this method opens up an exciting possibility to study IRE1 interactors and inhibitors, which may provide insights into the mechanistic underpinnings of IRE1-mediated ER stress homeostasis in plant tissues.

## Materials

Equipment used for the below-described experimentation is described in Supplementary Text. Details on the manufacturer and catalog numbers of the chemicals, consumables, reagents, and oligonucleotides used in the study are specified in Supplementary Table 1.

### Plant Material and Growth Conditions

Perform all the experiments with *Arabidopsis thaliana* Columbia-0 (Col-0) and wild-type *N. benthamiana* plants. Germinate the seeds on a super-fine germination mix under a 12 h light/12 h dark photoperiod (100 μmol/m^2^/s light intensity, 22°C, and 55% relative humidity). Individually transplant 2 weeks old *Arabidopsis* seedlings into round pots (3.5 × 3.5″) and water twice a week. Transplant 3 weeks old *N. benthamiana* seedlings into 6 × 4.5″ round pots and water once a week.

### Media, Reagents, Solutions, and Buffers

Prepare all solutions using ddH_2_0 and chemicals of analytical and molecular biology grade. When indicated, sterilize the solutions by autoclaving at 121°C for 30 min. All the preparations can be stored at room temperature unless specifically mentioned.

• Luria Bertani (LB): Prepare solid and liquid LB media following the steps described previously (Sambrook et al., 1989).
• Yeast Extract Beef (YEB): Prepare solid and liquid YEB media by dissolving 5 g Beef extract, 1 g Yeast extract, 5 g Peptone, 5 g sucrose, and 0.24 g MgSO_4_ in 1 L of ddH_2_0. To prepare solid YEB media, add 15 g/L Agar. Sterilize the prepared media by autoclaving.
• Antibiotics: Prepare the required 50 mg/ml antibiotic stocks in sterile Milli-Q water (when using Kanamycin Sulfate or Gentamicin Sulfate) or DMSO (when using Rifampicin). Dilute antibiotics to the final concentration of 50 μg/ml (1:1,000) in inoculation media or solid media plates.
• Protein Extraction Buffer: 50 mM HEPES (pH = 7.5), 150 mM NaCl, 5 mM EDTA (pH = 8.0), 5% glycerol, 2% PVPP, 0.01% NP-40, 5 mM DTT, 0.5 mM PMSF, 1× Plant protease inhibitor cocktail, 10 μM MG132. Sterilize HEPES by using vacuum filter/storage system (0.2 μm) and NaCl by autoclaving. DTT, plant protease inhibitor cocktail, 10 μM MG132, and PMSF must be stored at −20°C. Freshly prepare the buffer and set on ice while working with it.
• Immunoprecipitation buffer: Same as the protein extraction buffer without PVPP and DTT.
• Splicing buffer: 20 mM HEPES (pH = 7.5), 50 mM KOAc, 10 mM MgOAc, 1 mM DTT. Sterilize KOAc and MgOAc by using a vacuum filter/storage system (0.2 μm).
• Infiltration media: 10 mM MgCl_2_, 10 mM MES (pH 7.4), 200 μM Acetosyringone. Sterilize MgCl_2_ by autoclaving and MES by filter sterilization using a vacuum filter/storage system (0.2 μm). Acetosyringone should be stored in a −20°C freezer. Prepare the buffer freshly on the day of infiltration.

## Experimental Procedures

**FIGURE 1:**
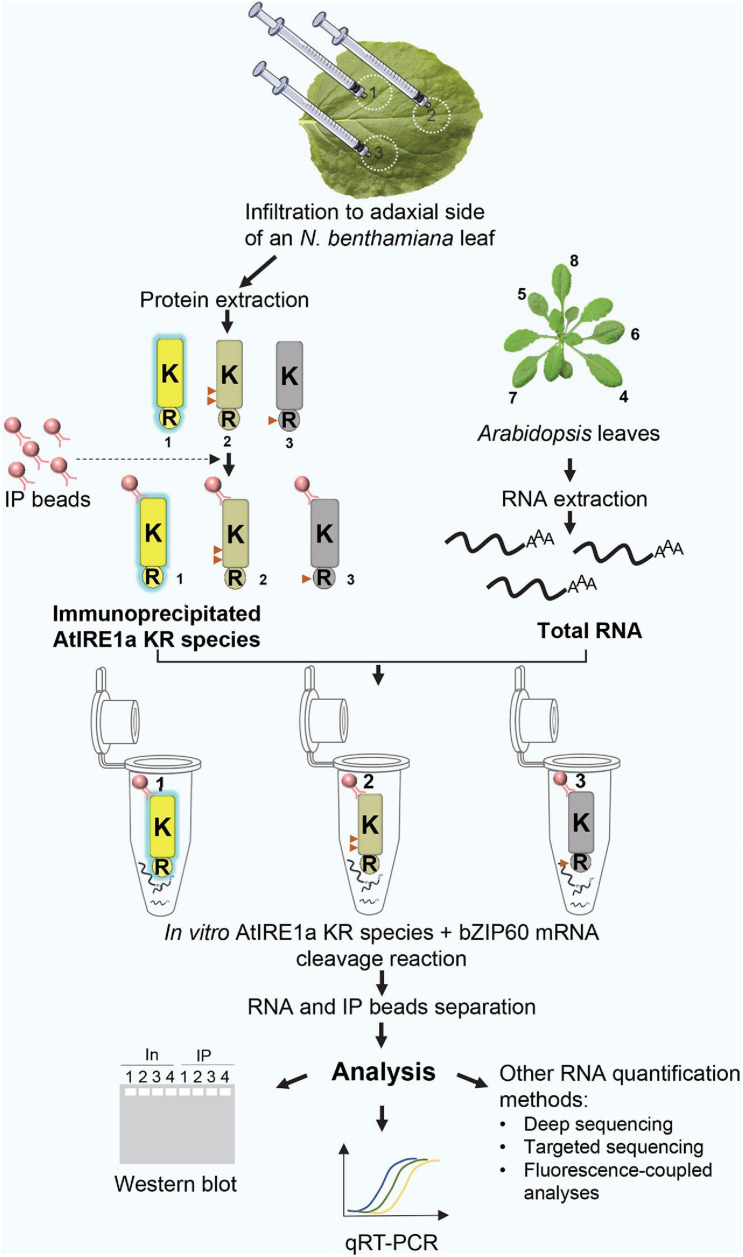
A schematic diagram representing an overview of the quantitative Arabidopsis IRE1a ribonuclease-dependent *in vitro* mRNA cleavage assay. Numbers 1, 2, 3 represent AtIRE1a-KR, AtIRE1a-KR phospho-dead, and AtIRE1a-KR RNase-dead variants, respectively. The active state of the protein is represented by yellow color and blue glow, the partially reduced activity is depicted in the gray-yellow shade, whereas the inactive state is represented using gray color and no glow. Red triangles on the proteins indicate the approximate positions of point mutations made to generate AtIRE1a-KR variants. Numbers 4, 5, 6, 7, 8 labeling Arabidopsis leaves represent the optimal leaf numbers to be harvested for RNA isolation.

A complete overview of the assay is shown in Figure 1.

### Step#1: Identification of Putative Residues Important for IRE1’s mRNA Cleavage Activity

**FIGURE 2:**
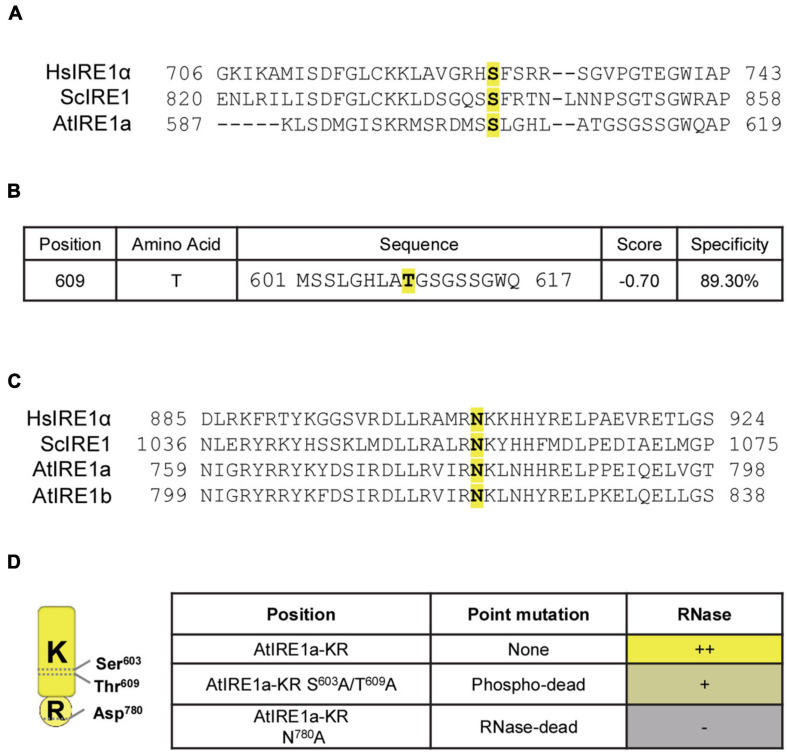
Identification of conserved amino acid sites important for AtIRE1a RNase function. **(A)** Multiple sequence alignment of IRE1 proteins. Partial sequences of *Homo sapiens* IRE1α (HsIRE1α), *Saccharomyces cerevisiae* IRE1 (ScIRE1), and AtIRE1a were aligned using Clustal Omega (https://www.ebi.ac.uk/Tools/msa/clustalo/). The conserved Serine residue is highlighted in yellow with bold font; **(B)** Prediction of plant-specific phosphorylation sites on AtIRE1a. The amino acid sequence of AtIRE1a was used to predict the phosphorylation sites using Musite (http://musite.sourceforge.net/). The conserved Threonine residues are highlighted in yellow with bold font; **(C)** Multiple sequence alignment of IRE1 proteins to map the conserved RNase domain residues. HsIRE1α, ScIRE1, AtIRE1a, and AtIRE1b partial protein sequences were aligned using Clustal Omega. The conserved Asparagine important for the IRE1 RNase activity is highlighted in yellow with bold font; **(D)** Conserved domain architecture of the Arabidopsis IRE1a KR protein domain, indicating critical amino acid residues and point mutations influencing the RNase activity. Relative levels of RNase activity detected in our experiments are indicated with (+) and (–) signs.

Inositol-Requiring Enzyme 1 is a dual-function stress transducer containing sequentially located Ser/Thr protein kinase and RNase domains, and its kinase activity is required for downstream RNase-mediated splicing of IRE1’s mRNA clients. It is well established that site-specific mutations in the human IRE1α kinase domain influence the activation and downstream RNase activity (Prischi et al., 2014). To evaluate whether our *in vitro* splicing assay can detect quantitative changes in the RNase activity of AtIRE1a (At2g17520), we scanned for potential Ser and Thr residues located within the AtIRE1akinase domain using the combination of Clustal Omega multiple protein sequence alignments (Sievers and Higgins, 2018) and the online Musite tool (Gao et al., 2010). We identified AtIRE1a’s Ser^603^ and Thr^609^ as candidate phosphorylation sites that are conserved within plant, yeast, and human IRE1 orthologs (Prischi et al., 2014; Figures 2A,B). Further, we sought to obtain an RNase-dead AtIRE1a variant. In a previous report on AtIRE1b, a substitution of Asp^820^ with Ala was shown to disable its RNA splicing activity but did not affect its kinase activity (Deng et al., 2013). We performed Clustal Omega multiple protein sequence alignments with human IRE1α, yeast IRE1, AtIRE1a, and AtIRE1b, and we identified Asp^780^ in AtIRE1a (corresponding to Asp^820^ of AtIRE1b) as a candidate for the critical residue affecting the RNase cleavage activity (Figure 2C). AtIRE1a’s Asp^780^ was also recently shown to be required for the full levels of canonical UPR gene expression (Hong et al., 2019). Consequently, we introduced point mutations to generate two AtIRE1a-KR variant constructs: a phospho-dead variant Ser^603^Ala + Thr^609^Ala, and an RNase-dead variant Asp^780^Ala (Figure 2D) to understand the direct contribution of these amino acids to the endonuclease activity of AtIRE1a *in vitro*.

### Step#2: Plant Tissue Sample Collection and Homogenization

Duration: 1 hour

(a) Select Arabidopsis leaves for RNA extraction: 4-week-old Arabidopsis leaves#4, 5, 6, 7, and 8 are ideal. The leaf numbers are indicated in Figure 1.
(b) Obtain *N. benthamiana* leaf tissue for protein extraction: harvest 250 mg of leaf tissue from the Agrobacterium-infiltrated area (see Step#5 below).
(c) Collect all samples in 2 ml free-standing screw cap tubes, each containing a 4 mm metal grinding bead. Freeze the samples in liquid nitrogen immediately following the harvest and homogenize them in a bead beater/homogenizer for 20 s at 25 Hz. Repeat 3 to 5 times until a fine powder of the leaf tissue is obtained. The frozen samples can be stored at −80°C for up to 1 week.

*Note*: *The samples should remain at cryogenic temperatures at all times. Make sure to re-freeze your sample multiple times during the homogenization cycles and pre-cool the homogenizer rack by submerging it in liquid nitrogen prior to grinding.*

### Step#3: Molecular Cloning and Mutagenesis of AtIRE1a Fragment Corresponding to the Coding Sequences of Kinase and RNase Domains (AtIRE1a-KR)

Duration: 6 days

(a) AtIRE1a-KR domain coding sequences PCR amplification and cloning

• Amplify AtIRE1a-KR CDS sequence using AtIRE1a-KR GW Stopless Forward and Reverse primers containing attB recombination sequences (Supplementary Table 1). In a 0.2 ml flat cap PCR tube set up a 50 μl reaction according to the instructions provided with Phusion High-Fidelity DNA Polymerase using 100 ng of Arabidopsis leaf cDNA library (concentration 140 ng/μl). The procedure for cDNA synthesis is described in Step#12 below. The PCR program is shown in Table 1.

**TABLE 1:** PCR cycling instructions for generating the AtIRE1a-KR PCR product.

Step	Cycles	Temperature	Time
Initial denaturation	1	98°C	30 s
Denaturation	30	98°C	10 s
Annealing		60°C	10 s
Extension		72°C	50 s
Final extension	1	72°C	7 min
Hold	1	4°C	∞

• Generate Gateway Entry clones: purify the product obtained from AtIRE1a-KR amplification reaction using the instructions provided in the user manual of the PCR gel purification kit. Perform Gateway BP reaction to recombine the IRE1a-KR amplicon into pDONR207 Entry vector using BP Clonase II enzyme mix and the instructions provided in the user manual (Invitrogen/Thermo Fisher Scientific).
• Transform 5 μl of the reaction in 50 μl of Top10 *Escherichia coli* competent cells.
• Plate the cells on Petri dishes containing LB solid media supplemented with Gentamicin. Incubate the inverted plates overnight in a 37°C incubator.
• Pick 10 colonies and inoculate them into liquid LB media supplemented with Gentamicin overnight in a 37°C shaker-incubator at 200 rpm.
• Perform miniprep. Subsequently, check for the presence of the AtIRE1a-KR insert by performing Sanger sequencing. Use BigDye Terminator1.1 cycle sequencing kit using pDONR207 Forward and pDONR207 Reverse primers, following the user instructions provided in the kit.

(b) Site-directed mutagenesis

• Based on the *in silico* analysis of AtIRE1a kinase and RNase domains described above (Step#1), perform site-directed mutagenesis of critical amino acids using the AtIRE1a-KR Entry clone to generate variants of AtIRE1a-KR. Set up a site-directed mutagenesis PCR reaction using Ser^603^Ala, Thr^609^Ala, and Asp^780^Ala substitution-specific primers (Supplementary Table 1) and Phusion High-Fidelity (HF) DNA Polymerase. The mutagenesis reaction setup and PCR cycling instructions are shown in Table 2 and Table 3. Set up all the reactions in 0.2 ml PCR tubes.

**TABLE 2:** AtIRE1-KR site-directed mutagenesis PCR setup.

Component	Volume
AtIRE1a-KR entry clone DNA	1 μl (50 ng)
5× Phusion^TM^ HF buffer	10 μl
10 μM mutagenesis forward primer	1 μl
10 μM mutagenesis Reverse primer	1 μl
10 mM dNTP mix	1 μl
Phusion^TM^ HF DNA Polymerase	0.5 μl
H_2_0	35.5 μl
Total	50 μl

**TABLE 3:** AtIRE1a-KR site-directed mutagenesis PCR cycling instructions.

Step	Cycles	Temperature	Time
Initial denaturation	1	98°C	5 min
Denaturation	30	98°C	50 s
Annealing		60°C	50 s
Extension		72°C	110 s
Final extension	1	72°C	7 min
Hold	1	4°C	∞

• After the PCR mutagenesis, set up a DpnI digestion reaction in a 0.2 ml flat cap PCR tube using the setup provided in Table 4. DpnI digests the methylated DNA, which allows for the elimination of the remaining non-mutagenized plasmid template.

**TABLE 4:** DpnI digestion reaction setup.

Component	Volume
PCR product	20 μl
Buffer Tango	6 μl
DpnI (2 U/μl)	2 μl
Nuclease-free water	32 μl
Total	60 μl

• Incubate the reaction overnight in a 37°C water bath. Transform 5 μl of the DpnI-digested product in 50 μl of Top10 *E. coli* competent cells. Plate the cells on LB solid media containing Gentamicin.
• Incubate the inverted plates overnight in a 37°C incubator. Pick 10 colonies and culture as described above in Step#3(a), perform miniprep, and check for the presence of mutation by performing Sanger sequencing as described in Step#3(a).

(c) Generating the expression clone

• Set up a Gateway LR recombination reaction using pDONR207: AtIRE1a-KR reference sequence or AtIRE1a-KR mutant variants [generated from Step#3(c)] and Gateway-compatible plant constitutive expression vector pGWB20: [35S: C-10xMyc] (Nakagawa et al., 2007) and Gateway LR Clonase II enzyme.
• Transform 5 μl of the reaction in 50 μl of Top10 *E. coli* competent cells.
• Plate the cells on LB solid media containing Kanamycin. Incubate the inverted plates overnight in a 37°C incubator.
• Pick 10 colonies, perform miniprep, confirm the presence of the insert, tag, start codon, stop codon, and reading frame by sequencing with primers AtIRE1a-K 1375 Forward and AtIRE1a-K 168 Reverse (Supplementary Table 1).

### Step# 4: *Agrobacterium* Transformation

Duration: 4 days

• Transform 5–7 μl (50–100 ng/μl) of miniprep-purified and sequence-verified expression clone plasmid DNA into50 μl freshly prepared *Agrobacterium tumefaciens* strain GV3101 competent cells using freeze-thaw transformation method (Weigel and Glazebrook, 2006). Recover the cells in 1 ml of liquid YEB media in a shaker-incubator maintained at 28°C and 200 rpm for 3 h.
• After incubation, pellet the cells and resuspend them in 100 μl of liquid YEB media. Plate the resulting cell suspension on YEB solid media plates supplemented with Gentamicin, Kanamycin, and Rifampicin. Incubate the inverted plates in a 28°C incubator for 3 days.
• Inoculate a single *Agrobacterium* colony transformant in a 14 ml round bottom tube containing 3 ml of liquid YEB media supplemented with Gentamicin, Kanamycin, and Rifampicin. Incubate the tubes in a shaker-incubator at 28°C and 200 rpm for 20 h.

### Step#5: Expression of AtIRE1a-KR and AtIRE1a-KR Mutant Variant Proteins

Duration: 2 days

• Pellet 3 ml of the overnight *Agrobacterium* culture at 4000 rpm for 10 min at room temperature. Wash the pellet twice with infiltration media. Dilute the resulting pellet to OD_600 *nm*_ = 0.5 with infiltration media. Incubate the cells with gentle agitation in the dark for 3 h.
• Use leaves of 6-week-old *N. benthamiana* plants for Agroinfiltration. Water *N. benthamiana* plants 3 h before infiltration. Poke a small hole on the adaxial side of the *N. benthamiana* leaf selected for Agroinfiltration. Using a slip tip syringe, infiltrate the pre-prepared *Agrobacterium* suspension by applying gentle pressure. Exert counter-pressure with a gloved finger on the other side of the leaf. Gently mark the dark appearing infiltrated area of the leaf using a permanent marker (Leuzinger et al., 2013).
• Wipe the excess culture on the leaf using Kimwipes. Place the plants under continuous light and the same growth conditions as mentioned above. Harvest the samples after 48 h as described above.

### Step#6: RNA Extraction

Duration: 1 hour

Perform RNA extraction using TRIzol reagent as follows. All the steps involved in the extraction process should be performed at room temperature unless mentioned otherwise.

• Add 1 ml of cold TRIzol reagent (maintained at 4°C) to the previously homogenized leaf tissue and mix well using a vortexer.
• Incubate the sample for 5 min and vortex intermittently to ensure complete mixing.
• Add 350 μl of chloroform and mix thoroughly using a vortexer.
• Centrifuge the lysate for 15 min at 12,000 rpm at 4°C. Note that after centrifugation, the mixture separates into two phases: a brown-colored lower phase containing debris and a colorless, aqueous upper phase.
• Using a micropipettor, transfer the colorless aqueous phase (200–400 μl) to a nuclease-free 1.5 ml tube containing 650 μl of isopropanol.
• Incubate for 2 h at −20°C.
• During the incubation time, continue with Step#7.

### Step#7: Purification of AtIRE1a-KR Wild-Type and AtIRE1a-KR Mutant Variant Proteins

Duration: 1 hour

• Add 250 μl of freshly prepared ice-cold lysis buffer to the sample and mix well by vortexing.
• Incubate the lysate on ice for 10 min and vortex intermittently to ensure complete mixing and lysis.
• Centrifuge the lysate for 25 min, 13,200 rpm at 4°C. Note that after centrifugation, three different layers should form: a dark-green aqueous layer on the top, a central thin white layer, and the bottom layer containing cellular debris.
• Transfer the top clarified supernatant layer, which contains the protein of interest, to a pre-chilled microcentrifuge tube. The volume of clarified supernatant should be approximately 200–230 μl.
• Save an aliquot (∼10 μl as an Input sample) of the AtIRE1a-KR and variant protein lysates to test the immunoprecipitation by Western blotting.

### Step#8: Immunoprecipitation

Duration: 30 minutes

Perform all centrifugation steps at 8,200 × *g* for 30 s at 4°C.

• Carefully mix the EZview Red Anti-c-Myc Affinity gel beads (Sigma Aldrich) by gently agitating until completely and uniformly suspended. Transfer 50 μl of bead slurry to a pre-chilled microcentrifuge tube and wash the beads using ice-cold 1 × PBS gently by tap mixing. Centrifuge and discard the supernatant using a micropipettor. Repeat the above-described wash step twice and set the tube with bead slurry on ice.
• Dilute the clarified supernatant with the Immunoprecipitation Buffer up to 0.5 ml and mix by vortexing for 10 s.
• Transfer the diluted extract to the tube containing the bead slurry. Set the tube on a rotator mixer for 2 h at 4°C.
• During the incubation time, continue with Step#9.

### Step#9: RNA Extraction Cont’d (Washing)

Duration: 1.5 hours

• After the incubation, centrifuge the mixture (from Step#6) for 10 min at 12,000 rpm at 4°C.
• Visually confirm the presence of total RNA precipitates forming a white pellet at the bottom of the tube. Carefully pipette out and discard the supernatant without disturbing the pellet.
• Wash the pellet by resuspending it in 1 ml of ice-cold 70% ethanol, briefly vortexing, then centrifuge for 10 min at 12,000 rpm at 4°C.
• Discard the supernatant using a micropipettor; repeat the wash step twice.
• Air-dry the RNA pellet for 10 min at room temperature; note that the pellet turns clear once dry.
• Resuspend the pellet in 30 μl of nuclease-free water by gently pipetting up and down several times.
• Measure the RNA concentration using a spectrophotometer. The expected RNA prep yield is up to 2,000 ng/μl.
• Set the isolated RNA on ice.

*Note: Start with multiple parallel RNA preps to ensure adequate concentration of your RNA sample for Step#11.*

### Step#10: Immunoprecipitation Cont’d:

Duration: 25 minutes

• After the incubation, centrifuge and discard the supernatant using a micropipettor.
• Wash the beads three times using the wash buffer by gently tapping the tube for 10 s.
• Save a small aliquot (∼10 μl as an IP sample) of the AtIRE1a-KR and variant protein immunoprecipitation beads for IP validation through Western blotting.
• Immediately proceed for Step#11. At this stage, the AtIRE1a-KR proteins should be bound on the immunoprecipitation beads, and ready for incubation with RNA.

*Note: To save time, immunoprecipitation beads can be washed and stored at 4°C for 12–16 h before the experiment.*

### Step#11: *In vitro* AtIRE1a-Dependent mRNA Cleavage Assay

Duration: 3 hours

• For each reaction, add the reaction mix shown in Table 5 to a 0.2 ml flat cap PCR tube. Set individual reactions for each AtIRE1a-KR wild-type and variants.

**TABLE 5:** *In vitro* AtIRE1a-dependent mRNA cleavage assay setup.

Component	Amount
Purified AtIRE1a KR beads	20 μl
Purified RNA	30 μg
Splicing buffer	Adjust the volume of reaction to 60 μl

• Incubate the tubes in a thermocycler for 3 h at 22°C.
• Remove the tubes from the thermocycler and centrifuge the tubes at 8,200 g for 1 min. Note that the reaction mixture should separate into two layers: the top clear supernatant containing RNA and the bottom containing EZview Red immunoprecipitation beads.
• Transfer approximately 30 μl of the top layer to a fresh 0.2 ml flat cap PCR tube.
• Measure the RNA concentration using a spectrophotometer and proceed for DNase treatment.

### Step#12: DNase Treatment and cDNA Synthesis

Duration: 2.5 hours

(a) Perform DNase treatment by setting up a reaction as shown in Table 6 in a fresh 0.2 ml flat cap PCR tube.

**TABLE 6:** DNase treatment reaction setup.

Component	Amount
RNA	10 μg
Turbo DNase buffer	2 μl
DNase enzyme	1 μl
Nuclease-free water	Adjust the volume of reaction to 20 μl

• Incubate the reaction mix in a thermocycler for 25 min at 37°C.
• Add 5 μl of DNase inactivating reagent, mix well by flicking the tubes several times, and incubate at room temperature for 5 min.
• Centrifuge the tubes for 1 min at 10,000 rpm at room temperature. The reaction mixture should separate into two layers, a clear top layer containing DNA-free RNA and the bottom white layer.
• Transfer the top layer to a fresh PCR tube and proceed to reverse transcription reaction.

(b) Perform reverse transcription by setting up a reaction as shown in Table 7 in a fresh 0.2 ml flat cap PCR tube.

**TABLE 7:** Reverse transcription reaction setup.

Component	Amount
RNA	7 μl
10 mM dNTPs	1 μl
Oligo dT Primers	0.5 μl
Total	8.5 μl

• Incubate the reaction mixture in a thermocycler for 5 min at 65°C.
• Chill the reaction tubes on ice for 1 min and add the cDNA synthesis mix shown in Table 8.

**TABLE 8:** cDNA synthesis mix.

Component	Volume
DTT	2 μl
Reverse transcriptase buffer	4 μl
Reverse transcriptase enzyme	0.25 μl
Nuclease-free water	5.25 μl
Total	11.5 μl

• Mix by pipetting gently up and down, then centrifuge at 10,000 rpm for 10 s. Incubate the reaction mixture in a thermocycler at 42°C for 60 min, followed by 72°C for 20 s.
• Stopping Point: store the prepared cDNA at −20°C if desired. Proceed with Step#13 within the next week.

### Step#13: qRT-PCR

Duration: 2 hours

• Dilute the synthesized cDNA using nuclease-free water to achieve a 1:8 dilution ratio.
• Dilute the primers to 10 μM concentration. Use primers according to the gene target(s) and controls as shown in Table 9.

**TABLE 9:** Target genes and controls used for qRT-PCR.

Gene target/controls	Purpose
*AtbZIP60un*	To measure unspliced bZIP60 mRNA
*AtbZIP60s*	To measure spliced bZIP60 mRNA
*UBQ5* (Arabidopsis Ubiquitin 5)	Housekeeping gene to normalize RNA expression
Empty vector control	A control to measure basal levels of unspliced bZIP60 mRNA
Negative control	No RNA

• Prepare a qRT-PCR master mix by mixing the reaction components shown in Table 10 (the reaction mixture should be prepared on ice and protected from bright light).

**TABLE 10:** qRT-PCR master mix.

Component	Volume
Go Taq qPCR master mix	5 μl
Forward primer	1 μl
Reverse primer	1 μl
cDNA	3 μl
Total	10 μl

• Add 3 μl of the cDNA template and 7 μl of the qRT-PCR master mix to each individual well of the nuclease-free qPCR reaction plate.
• Seal the wells using a qRT-PCR sealing film and spin down the plate briefly to collect the content of the wells to the bottom. Perform the qRT-PCR using the cycling instructions provided in Table 11.

**TABLE 11:** qRT-PCR cycling instructions.

Step	Cycles	Temperature	Time
Initial denaturation	1	98°C	10 min
Denaturation	40	98°C	15 s
Annealing		55°C	30 s
Extension		72°C	30 s

### Step#14: Western Blotting

Duration: 9 hours

Western blotting analyses follow the protocols described by our laboratory previously (Liu et al., 2019).

**FIGURE 3:**
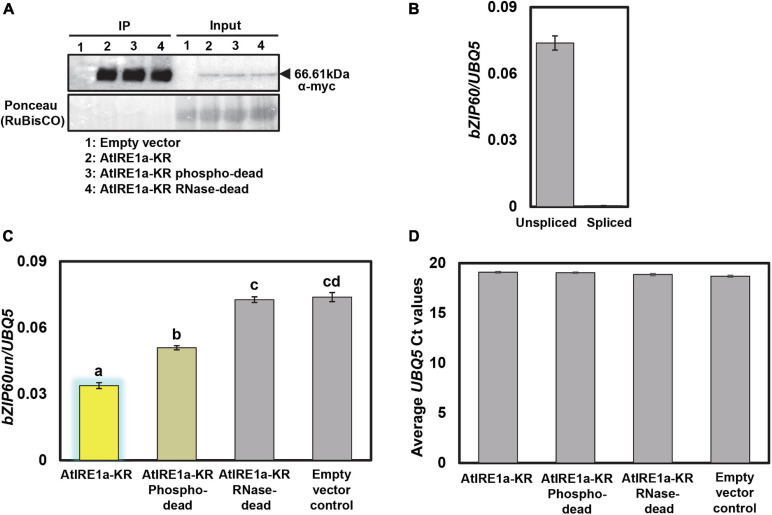
Protein expression levels of *N. benthamiana* expressed AtIRE1a-KR variants and their effects on *AtbZIP60* mRNA cleavage. **(A)** 10myc-tagged AtIRE1a-KR, AtIRE1a-KR phospho-dead, and AtIRE1a-KR RNase-dead proteins expressed in *N. benthamiana* were subjected to immunoprecipitation. A Western blot probed with an anti-myc antibody was used to detect the presence of protein. Ponceau staining in the input fraction indicates equal amounts of proteins expressed; **(B)** Bar graphs representing *bZIP60us* (unspliced bZIP60) and *bZIP60s* (spliced bZIP60) mRNA levels before performing the *in vitro* mRNA cleavage assay; **(C)** Bar graph representing *bZIP60us* mRNA levels after performing the *in vitro* mRNA cleavage assay. The measured mRNA expression levels were normalized to an Arabidopsis housekeeping gene *UBQ5* (*Ubiquitin 5*). The colors of the bars correspond to the RNase status as indicated above (Figure 2D). Statistical analysis was performed using an online interface (https://astatsa.com) by one-way ANOVA with *post-hoc* Tukey HSD test calculator. Error bars show mean ± SD. Bars labeled by different letters differed from each other at *p* < 0.05; **(D)** The levels of *UBQ5* transcripts in various samples were monitored to control for mRNA degradation. Error bars show mean ± SD and differences were not statistically significant. All experiments were performed at least three times, each with three independent technical replications.

• Prepare the input sample from Step#7 by adding 2.5 μl of 4× sample buffer and IP sample by adding 10 μl of 1× PBS and 4 μl of LDS (Lithium dodecyl sulfate) sample buffer (4×) to the 10 μl of bead slurry.
• Boil the sample for 10 min at 95°C on a heating block.
• Separate the protein samples on an 8% SDS-PAGE at 80 V for 30 min. Next, increase the voltage to 100 V and continue the electrophoresis until the dye front runs out of the bottom of the gel.
• Transfer the proteins onto a nitrocellulose membrane with a wet electroblotting system.
• Detect the myc epitope-tagged wild-type AtIRE1a-KR and AtIRE1a-KR variants in input and IP by incubating membranes with primary anti-Myc antibody for 2 h at room temperature. After incubation with the primary antibodies, incubate the membrane with a secondary anti-mouse HRP-conjugated antibody for 1 h at room temperature.
• Detect the immunoblots using Cytiva Amersham^TM^ ECL^TM^ western blot detecting reagents (Figure 3A).

## Results

A successful *in vitro* AtIRE1a-dependent mRNA cleavage assay is expected to show a difference in unspliced levels of the selected mRNA read-out transcript compared to controls. We employed purified recombinant myc-tagged wild-type AtIRE1a-KR protein fragments in our *in vitro* splicing assay along with empty vector control. As in every quantitative assay involving purified peptides, it is crucial to control for equal amounts of the AtIRE1a-KR proteins used in this assay. Toward this, we first confirmed the accumulation levels and the expected size of the protein fragments using Western blotting with α-myc antibodies (Figure 3A). We confirmed that the mRNA of unspliced bZIP60 (*bZIP60un*), a *bona fide* AtIRE1a splicing target, could be readily quantified in the total RNA samples extracted from Arabidopsis leaves prior to the *in vitro* splicing assay while no measurable accumulation of spliced bZIP60 (*bZIP60s*) could be detected using a *bZIP60s*–specific primer set (Figure 3B). Subsequently, we performed the *in vitro* splicing assay by co-incubating total Arabidopsis leaf RNA with AtIRE1a-KR or empty vector control and subsequently measuring the *bZIP60un* levels by qRT-PCR analysis using a *bZIP60un*–specific primer set. Compared to vector control, we showed ∼54% reduction in the levels of *bZIP60un* mRNA indicating that the purified AtIRE1a-KR is efficacious in cleaving the *bZIP60un* transcript (Figure 3C). When we tested the AtIRE1a-KR RNase-dead variant, we detected a statistically insignificant reduction (∼1.5%) of *bZIP60un* compared to the empty vector sample, which confirmed that the Asp^780^Ala mutation abolishes the RNase activity of AtIRE1a *in vitro*, similar to the Asp^820^Ala mutation in AtIRE1b (Deng et al., 2013). The third AtIRE1a construct tested, the phospho-dead Ser^603^Ala + Thr^609^Ala variant, processed 1.5 fold less *bZIP60un* than the wild-type AtIRE1a-KR, showing a ∼31% reduction compared to the empty vector sample (Figure 3C). Thus, we confirmed the importance of these two residues for full AtIRE1a-KR splicing efficacy. Finally, to check for possible RNA degradation levels in all samples/reactions, we quantified and plotted the levels of the *UBQ5* reference gene and we demonstrated that no detectable RNA degradation was observed within the sample groups (Figure 3D).

## Discussion

We established a novel quantitative *in vitro* cleavage assay that combines recombinant AtIRE1a protein fragments and total RNA isolated from Arabidopsis leaves to determine *bZIP60us* mRNA cleavage efficiency. Previously, we and others have measured Arabidopsis, maize, potato, or tomato IRE1’s RNase output in plant cells using bZIP60 mRNA splicing assays, utilizing both semi-quantitative RT-PCR agarose gel-based and quantitative, qRT-PCR-based methodologies (Deng et al., 2011, 2013; Nagashima et al., 2011; Moreno et al., 2012; Zhang et al., 2015; Gaguancela et al., 2016; Afrin et al., 2020b; Li et al., 2020; Kaur and Kaitheri Kandoth, 2021). While useful, these techniques do not allow precise time-resolved measurements of RNA processing by IRE1, and can only capture the splicing/degradation executed by the endogenous, wild-type versions of the IRE1 proteins. Moreover, the single *ire1a* or *ire1b* loss-of-function mutations in Arabidopsis only cause a partial depletion of the *bZIP60* splicing activity, likely due to the compensatory mechanism exerted by the other paralog(s) (Deng et al., 2011; Nagashima et al., 2011; Moreno et al., 2012), making it impossible to study the contributions of the individual IRE1 proteins to the mRNA substrate cleavage.

Similar limitations have been recently recognized in the animal systems, which yielded the development of an *in vitro*-based human IRE1α cleavage assay (Acosta-Alvear et al., 2018; Karagoz et al., 2019). Similar to our method, this protocol also utilizes the cytosolic domain fragment of IRE1α and a well-established heterologous protein expression host (the SF21 ovarian cells from a moth *Spodoptera frugiperda*). In contrast to our assay that uses total leaf RNA preparations, the human IRE1α splicing assay is executed with specific lab-synthesized RNA templates corresponding to known or presumed IRE1 targets. While not necessary under our conditions, we recognize that such an experimental modification could expand the applications of our assay for quantitative testing of any novel, yet-to-be-discovered plant IRE1 substrates. In particular, RIDD target mRNAs could be tested and their mutant versions could be engineered to be cleavage- and degradation-resistant, thus proving the roles of nucleotides essential for the splicing specificity. Such modifications, if adopted, would also require a different method of the RNA substrates quantification because the standard oligo d(T)-based reverse transcription procedures wouldn’t be applicable due to the lack of the poly(A) tails on the RNA molecules under study. One possible solution to circumvent this could be TBE-urea PAGE gel electrophoresis and ImageJ-based quantification of the RNA substrate bands (Karagoz et al., 2019).

The value of an *in vitro*-based IRE1 splicing assay in plants was first recognized a decade ago when Deng et al. (2011) performed initial investigations of the AtIRE1a and AtIRE1b RNase activity. In their qualitative assay, a 125b-long fragment of *bZIP60us* mRNA was used as substrate. His-tagged, C-terminal fragments of AtIRE1a and AtIRE1b were synthesized in *E. coli* and partially purified prior to the splicing assay. Their splicing activity was tested and confirmed *in vitro* by PAGE gel electrophoresis followed autoradiography-mediated detection of discrete bands corresponding to shorter RNA fragments. Further, Deng et al. (2013) employed that technique to evaluate a set of mutations within AtIRE1b kinase and RNase domains, and determined that Ser^820^ is necessary for the RNase activity; a discovery that helped guide our AtIRE1a mutagenesis approach. The gel-based detection and quantification of cleavage mRNA products could be combined with our qRT-PCR quantification to allow for more precise and robust monitoring of the IRE1-mediated splicing activity.

Given the limitations of currently available genetic and biochemical approaches, we set out to develop a robust assay that allows for precise and quantitative measurements of IRE1’s RNase activity in the test tube without the need for substrate synthesis and labeling. Instead, we have shown that total Arabidopsis leaf RNA, isolated through a standard TRIzol protocol, can be efficiently spliced by AtIRE1a under *in vitro* conditions. This technical advance over singular pre-synthetized RNA substrates streamlines the experimental design, allows simultaneous monitoring of more than one substrate, and facilitates the subsequent quantification steps by utilizing the popular qRT-PCR technique, well established in many laboratories. Furthermore, the AtIRE1a protein fragments used in our assay were expressed in a plant-based *N. benthamiana* system, which presents an advantage over *E. coli*-produced proteins. The plant expression host is known to produce correctly folded recombinant proteins equipped with plant-specific post-translational modifications, translating into more accurate experimental findings (Bally et al., 2018). Further, our results suggest that the AtIRE1a homodimerization, at least within its C-terminal domain, may not be required for the RNase activity and execution of cytoplasmic splicing, as indicated by the presence of monomeric AtIRE1a-KR in Figure 3A. It cannot, however, be excluded that the AtIRE1a-KR monomers can form dimers and/or oligomers during the subsequent *in vitro* splicing step of our method. Moreover, we show that the *in vitro* splicing can occur in the absence of a cognate RNA ligase and this process does not require the native ER membrane microenvironment, although it is important to recognize that its absolute efficiency might differ from the findings recorded *in planta*.

While the advent of *in vitro* systems has massively promoted our understanding of molecular and biochemical mechanisms governing cellular signaling, a number of limitations have been acknowledged. *In vitro* assays are admittedly restricted in the information they can provide about the regulatory differences between different cell/tissue types, and can’t faithfully replicate the local cellular microenvironments, where hundreds of genes are up- or down-regulated, and additional metabolites and proteins can interact to alter the system’s homeostasis. Thus, we recognize that the relative *bZIP60us in vitro* cleavage efficiencies reported here (Figure 3C) are likely lower than the endogenous *in planta* splicing levels; nonetheless, they provide a good and reproducible approximation of each AtIRE1a variant’s ability to process its mRNA target. While some of the previously published human IRE1α cleavage assays used recombinant proteins with their epitope tags subsequently removed (Acosta-Alvear et al., 2018; Karagoz et al., 2019), we and others found that this step was not necessary for the AtIRE1a and AtIRE1b splicing function (Deng et al., 2011, 2013), albeit it is plausible that the presence of the tag has affected the cleavage efficiency to some extent.

Among the possible future applications of our method are studies on IRE1 inhibitors. Human IRE1α was shown to undergo an inhibition of its RNase activity in the presence of various chemicals, such as salicylaldehydes, MKC-3946, MKC-9989, OICR464, OICR573, toyocamycin, STF-083010, and 4 μ8C (Jiang et al., 2015). Interestingly, lysine 907 in HsIRE1α that was shown to be the target residue of STF-083010 and 4 μ8C (Papandreou et al., 2011; Cross et al., 2012), is conserved in the Arabidopsis IRE1 homologs, making it a plausible target to be tested experimentally. Conversely, it was shown that a flavonoid compound quercetin promotes the yeast IRE1’s and human IRE1α’s RNAse activity by binding to the “Q site” in the RNase domain (Wiseman et al., 2010; Jiang et al., 2015). While the role of quercetin in the regulation of plant IRE1 homologs remains unknown, it could be readily tested using the assay described here.

Further, the above-described assay could be employed in additional IRE1 structure-function assays, for example, coupled with phosphatase treatments for testing the functional importance of putative phosphorylation sites, or supplementation with RNase inhibitors when assaying novel residues potentially involved in the RNA cleavage activity. Finally, for splicing-specific assays, it may be feasible to test if the addition of an RNA ligase to the reaction mix could result in joining the ends of bZIP60 mRNA in Arabidopsis or its homologs in other plants. While it is known that AtbZIP60 is ligated with the help of a tRNA ligase RLG1 *in vivo* (Nagashima et al., 2016), it remains to be tested if the same process could be replicated under *in vitro* conditions, and if so, whether other tRNA ligases could catalyze this reaction, such as the mammalian RtcB that ligates the XBP1 mRNA (Lu et al., 2014).

In summary, this AtIRE1a RNase-dependent mRNA cleavage assay can be employed in a variety of laboratories studying plant ER stress to answer a wide range of biological questions including mechanistic studies on Arabidopsis UPR, IRE1 mutagenesis and structure-function studies, validation of novel RIDD targets, and investigations of the IRE1-bZIP60 axis in other plant species including crops.

## Limitations

• This method provides a quick and robust method to screen potential targets of ER.
• Stress sensor AtIRE1a; however, the splicing/degradation is performed in a non-native state of the protein that can affect its RNase activity levels.
• There could be variation in bZIP60 mRNA levels in the collected RNA samples between the experimental replicates. Therefore, it is recommended to perform multiple replicates to identify a trend and include appropriate controls to estimate the accuracy.
• This method measures a decline of *bZIP60un* mRNA as a proxy for AtIRE1a RNase cleavage activity levels. It cannot be used to measure the native product of this splicing reaction, bZIP60s, because the cognate tRNA ligase RLG1 is missing from the reaction (Nagashima et al., 2016). Therefore, it is necessary to monitor for mRNA degradation as illustrated in Figure 3D.
• This method is not suitable for advanced analyses pertaining to kinetic or pharmacokinetic characterization of IRE1 catalytic activity; however, it is sufficiently sensitive to be used in preliminary structure-function analyses of plant IRE1 homologs before advancing into *in planta* stable transgenic approaches.

## Troubleshooting

### Problem 1: Low/No Protein Yield

#### Potential Solution

Although protein immunoprecipitation is a routine technique, there are some limitations when working with plant samples.

• The age and health of the *N. benthamiana* leaves are principal factors influencing the protein yield. Please follow the recommended growth conditions and watering schedule.
• The temperature during homogenization and protein preparation is critical for optimal protein yield. The plant material during homogenization must be maintained at an ultra-cold temperature to obtain a finely pulverized sample. The sample after homogenization should be stored in a −80°C deep freezer until the day of the experiment (but no longer than 2 weeks after harvesting). All buffers and reagents used for protein preparation must be refrigerated and should be kept on ice during experiments.
• Harvesting the sample after 48 h of agroinfiltration is necessary to obtain higher protein levels. During the development of this protocol, we performed a systematic time-course characterization of the transient AtIRE1a-KR protein expression patterns in *N. benthamiana* leaf tissue and we found the 48 h post-infiltration to be the optimal time point.

### Problem 2: Lower RNA Yield Than Expected

#### Potential Solutions

• A lower RNA yield may indicate that the sample was not homogenized properly. Please repeat the above-described steps of RNA extraction, carefully following all instructions.
• Perform thorough RNase decontamination of your working environment before you proceed with the RNA prep. Use RNaseZAP RNase decontamination solution or a similar product.
• Be careful while decanting the 70% ethanol during the RNA washing stage, as the RNA pellet is very delicate and can detach from the tube’s bottom.
• Make sure that the RNA pellet is completely dry before resuspending it (ensure that the pellet has become completely colorless after drying).

### Problem 3: No Difference in the Unspliced Levels of Selected mRNA Transcript as Compared to Control

#### Potential Solutions

• Immunoprecipitated AtIRE1a-KR and AtIRE1a-KR mutant variants should immediately be incubated with the purified RNA. Delay in the incubation may influence the mRNA cleavage activity of AtIRE1a-KR; therefore, it is highly recommended to perform Steps#6–12 uninterruptedly within 1 day.
• The presence of non-specific proteins may interfere with the mRNA cleavage activity of AtIRE1a-KR. The bead washing step after immunoprecipitation is crucial to get rid of any unwanted proteins.
• Similar protein contaminants can also be introduced by the RNA component. Carefully pipetting the supernatant during Step 6 ensures no protein carryover.
• Washing the RNA pellet carefully during Step#9 is key to obtain purified RNA without the presence of inhibitory phenolics and other undesired and potentially inhibitory compounds. Additional washes can be performed if required.

## Data Availability Statement

The raw data supporting the conclusions of this article will be made available by the authors, without undue reservation.

## Author Contributions

XL, DD, and KP-M designed the splicing assay. DD and XL prepared all constructs described in this study, performed *in vitro* splicing assay experiments, and analyzed the data. CA assisted in the experiments. DD and KP-M analyzed the data and wrote the manuscript. KP-M coordinated the research program and oversaw the experimental work. All authors discussed the results, critically reviewed the manuscript, and provided feedback.

## Conflict of Interest

The authors declare that the research was conducted in the absence of any commercial or financial relationships that could be construed as a potential conflict of interest.

## Supplementary Material

Supplementary Table 1 Details on the manufacturers and catalog numbers of the chemicals, consumables, reagents, and oligonucleotides used in the study.

Supplementary Text Description of equipment used for the below-described experimentation.
