# Supplementary material for: A Quantitative Arabidopsis IRE1a Ribonuclease-Dependent in vitro mRNA Cleavage Assay for Functional Studies of Substrate Splicing and Decay Activities
Source: Front Plant Sci. 2021 Jul 20;12:707378. doi: 10.3389/fpls.2021.707378 (PMC8329651; doi:10.3389/fpls.2021.707378)
Supplement: Supplementary Table 1 — Details on the manufacturers and catalog numbers of the chemicals, consumables, reagents, and oligonucleotides used in the study. [file Data_Sheet_1.DOCX]

***Supplementary Information***

**Table S1:** Details on manufacturers and catalog numbers of the chemicals, consumables, reagents, and oligonucleotides used in the study.

| REAGENT or RESOURCE | SOURCE | IDENTIFIER |
| --- | --- | --- |
| Antibodies and Immunoprecipitation beads | | |
| c-Myc antibody (9E10) | Santa Cruz Biotechnology | Sc-40 |
| Goat anti- Mouse IgG (Kappa light chain) secondary antibody, HRP | Invitrogen | PA1-86015 |
| EZview Red Anti-C-Myc Affinity Gel | Millipore Sigma | E6654 |
| Bacterial Strains | | |
| *E. coli* strain OneShot TOP10 | ThermoFisher | C404003 |
| *E. coli* strain DB3.1 | ThermoFisher | 11782-018 |
| *Agrobacterium tumefaciens* strain GV3101 | Gold Biotechnology | CC-207-A |
| Chemicals, and Enzymes | | |
| LB Broth, Miller | Fisher BioReagents | BP 1426-500 |
| Yeast Extract | Bio Basic | MD19 |
| BBL Beef Extract Powder | BD | 212303 |
| Bacto Proteose Peptone | BD | 211684 |
| Magnesium Sulfate Anhydrous | Fisher Chemical | M65-500 |
| D- Sucrose | Fisher BioReagents | BP220-212 |
| Agar, pure, powder | Acros Organics | AC400402500 |
| Kanamycin Sulfate | Bio Basic | KB0286 |
| Gentamycin Sulfate | Bio Basic | GB0217 |
| Rifampicin | Bio Basic | 13292-46-1 |
| HEPES (Free Acid) | Bio Basic | 7365-45-9 |
| Sodium Chloride (NaCl) | Bio Basic | 7647-14-5 |
| Ethylenediamine tetra acetic acid (EDTA), Disodium salt Dihydrate | Fisher Chemical | S312-500 |
| Glycerol | Bio Basic | 56-81-5 |
| Poly(vinylpolypyrrolidone) (PVPP) ~110µm Particle Size | Fluka Analytical | 77627 |
| NP-40 Surfact- Amps detergent solution | Thermo Scientific | 85124 |
| DTT 1,4-Dithiothreitol | Millipore Sigma | 11583786001 |
| Phenylmethylsulfonyl Fluoride (PMSF) | Roche | 10837091001 |
| Protease Inhibitor Cocktail for plant cell and tissue extracts | Millipore Sigma | P9599 |
| MG-132, Ready Made Solution ≥90% (HPLC) | Millipore Sigma | M7449 |
| Potassium Acetate (KOAc) | Bio Basic | 127-08-2 |
| Magnesium Acetate (MgOAc) | Bio Basic | 16674-78-5 |
| Magnesium Chloride (MgCl_2_) | Acros Chemicals | 223210010 |
| 2-(N-morpholino)ethanesulfonic acid (MES) Monohydrate, ultrapure | Thermo Scientific | J18886-22 |
| Gateway BP clonase II enzyme mix | Invitrogen | 11789021 |
| Gateway LR clonase II enzyme mix | Invitrogen | 11791019 |
| Phusion High- Fidelity DNA polymerase 2U/µl | Thermo scientific | F530-Xl |
| *dpn1* (10U/µl) | Thermo Scientific | ER1701 |
| Immobilon-P Transfer membrane 0.45µm | Merck Millipore | IPVH00010 |
| NuPAGE LDS sample buffer (4X) | Invitrogen | NP0007 |
| RNaseZap cleaning agent for removing RNase | Millipore Sigma | R2020 |
| Invitrogen Trizol Reagent | Fisher Scientific | 15-596-018 |
| Kits | | |
| EZ10 spin column PCR product purification kit | Bio Basics | BS363 |
| TURBO DNA-*free* Kit | Invitrogen | AM1907 |
| GoTaq qPCR Master Mix | Promega | A6002 |
| Amersham ECl Western Blotting Detection Reagent | Cytiva | RPN2109 |
| Oligonucleotides (sequence 5’ -> 3’) | | |
| AtIRE1a-KR GW stopless Forward  GGGGACAAGTTTGTACAAAAAAGCAGGCTCCATGAAAAAGTTTTCGTCGAGGGGCAGTG | Eurofins Genomics | NA |
| AtIRE1a-KR GW stopless Reverse  GGGGACCACTTTGTACAAGAAAGCTGGGTCGATGATGTCGCATTTGAAGTACTTTC | Eurofins Genomics | NA |
| AtIRE1a-KR S^603^A + T^609^A Forward  CTGCCAGAACCTGCGGCAAGGTGACCC | Eurofins Genomics | NA |
| AtIRE1a- KR S^603^A + T^609^A Reverse  GGGTCACCTTGCCGCAGGTTCTGGCAG | Eurofins Genomics | NA |
| AtIRE1a-KR N^780^A Forward  GTTACGAGTCATCAGGGCCAAACTGAATCATCATC | Eurofins Genomics | NA |
| AtIRE1a-KR N^780^A Reverse  GATGATGATTCAGTTTGGCCCTGATGACTCGTAAC | Eurofins Genomics | NA |
| AtbZIP60un Forward  GGAGACGATGATGCTGTGGCT | Eurofins Genomics | NA |
| AtbZIP60un Reverse  CAGGGATTCCAACAAGAGCACAG | Eurofins Genomics | NA |
| AtbZIP60s Reverse  CAGGGAACCCAACAGCAGACT | Eurofins Genomics | NA |
| UBQ5 Forward  GACGCTTCATCTCGTCC | Eurofins Genomics | NA |
| UBQ5 Reverse  GTAAACGTAGGTGAGTCC | Eurofins Genomics | NA |
| pDONR207 Forward  TCGCGTTAACGCTAGCATGGATCTC | Eurofins Genomics | NA |
| pDONR207 Reverse  GTAACATCAGAGATTTTGAGACAC | Eurofins Genomics | NA |
| IRE1a-K 1375 Forward  CTCATTGCATCAGACCAACAC | Eurofins Genomics | NA |
| IRE1a-K 168 Reverse  CCTCCTTCAATGAGCTCGAA | Eurofins Genomics | NA |
| Promega oligo (dT) primers | Promega | PR-C1101 |
| Recombinant DNA | | |
| Plasmid: pDONR207 | Invitrogen | 12213-013 |
| Plasmid: pGWB21 | Nakagawa et al. | AB289784.1 |
| Protein sequences | | |
| AtIRE1a and AtIRE1b protein and CDS sequences | TAIR | At2g17520, At5g24360 |
| HsIRE1a protein sequence | NCBI PDB | NP_001424.3 |
| ScIRE1a protein sequence | SGD | YHRO79C |
| Other consumables | | |
| Twin.tech Real-Time PCR Plate 96, skirted | Eppendorf | 951022015 |
| Adhesive Sealing Sheets | Thermo Scientific | AB-0558 |
| BD slip tip sterile syringe | Fisher Scientific | 14-823-434 |
| Kimwipes | Fisher Scientific | 06-666-11 |
| Fisher brand 2ml free standing screw cap tubes natural | Fisher Scientific | 02-681-343 |
| Thermo scientific screw cap microcentrifuge tube caps | Fisher Scientific | 14-755-248 |
| 4mm carbon steel ball bearing G1000 | BC Precision | NA |
| 2.8cf Fafard Superfine germination mix | Sungrow | FAFGERM |
| Large round pots (6′′ X 4.5′′) | Landmark plastics | LMIR600MGGR |
| Small round pots (4′′ X 3.5′′) | Landmark plastics | LMX4RMGTGR |

**Supplementary Text:**

**Equipment**

- Laboratory standard refrigerated centrifuge (Fisher Scientific Microspin 17R, 13- 100-675) and accompanying rotor (75003524)
- Laboratory standard centrifuge (Fisher Scientific Microspin 75002461) and accompanying rotor (75003524)
- Benchtop mini- centrifuge (Fisherbrand mini- centrifuge, 12-006-901)
- Bead Mill Homogenizer (OMNI Bead Ruptor 96 SKU: 27-0001) with accompanying screw cap tube holders (27-106)
- Thermocycler (Eppendorf Mastercycler X50s, EP6311000010)
- Quantitative Real-Time PCR equipment (BioRad CFX 96, 1855200)
- Laboratory freezers, -20°C under-counter refrigerator (Thermo Scientific TSX505) and -80°C ultra low-temperature freezer (Thermo Scientific FORMA 700 series, 73-20A)
- Benchtop vortex mixer (Fisher Scientific Std vortex mixer, 02215365)
- Benchtop Tube rotator (Thermo Scientific Tube revolver rotator, 88881001) with accompanying paddles.
- Mini benchtop shaker incubator (Thermo Scientific MaxQ4450 )
- Spectrophotometer (Eppendorf Biophotometer plus, 613ZJ102916)
- Heating cooling dry bath (Thermo Scientific, 88880029)
- Protein electrophoresis and electroblotting system (BioRad Mini-ProteanTetra electrophoresis system, The Mini Trans-Blot Module 1703930, 1703935)

### Manual Single-Channel Micropipettes (Eppendorf Research plus 0.1–2.5, 2-20, 20-200 and 100–1,000 µL, 2231000773 )
